# Research on the effectiveness of CMA and WES results in pregnant females with US findings and normal karyotype results from conventional karyotype analysis

**DOI:** 10.1515/crpm-2024-0049

**Published:** 2025-06-09

**Authors:** Masum Kayapınar, Zafer Bütün, Ece Akça Salık, Sinem Kocagil, Ezgi Susam

**Affiliations:** Department of Perinatology, Private Clinic, Mersin, Türkiye; Department of Perinatology, Private Clinic, Eskişehir, Türkiye; Eskişehir City Hospital, Obstetrics and Gynecology, Eskişehir, Türkiye; Eskişehir Osmangazi University, Genetic, Eskişehir, Türkiye; Sakarya Research and Education Hospital, Genetic, Sakarya, Türkiye

**Keywords:** whole-exome sequencing (WES), chromosomal microarray analysis (CMA), conventional karyotype, next-generation sequencing (NGS)

## Abstract

**Objectives:**

With the advancement of next-generation sequencing (NGS), whole-exome sequencing (WES) has proven useful in diagnosing various diseases, particularly neurodevelopmental disorders, during both the prenatal and postnatal periods. In this study, we examined the correlation between the results of chromosomal microarray analysis (CMA) and WES in pregnant women, as compared to conventional karyotype analysis and ultrasound (US) findings.

**Methods:**

Fetal US were performed on pregnant females referred to our clinic with suspected fetal anomalies, as well as those who had anomalies detected by targeted US. Comprehensive counseling was provided to all parents. Karyotyping, CMA, and WES were offered for all fetuses through amniocentesis, CVS, and cordocentesis. We compared the demographic data and ultrasound findings of pregnant females with normal and abnormal WES results.

**Results:**

WES results indicated a normal karyotype in 14 pregnant females and an abnormal karyotype in 12 pregnant females. CMA showed an abnormal karyotype in three of the pregnant females, whose WES results were normal. US findings were more frequently observed in pregnant females with abnormal WES results.

**Conclusions:**

Among the organ systems, the cardiac system is at the highest risk for anomalies. The risk further increases when multiple system anomalies are present. Incorporating WES alongside CMA may enhance diagnostic accuracy and be beneficial for subsequent pregnancies. Our US results do not align with the existing literature, and further evaluations with larger patient populations are needed to reconcile these findings with current research.

## Introduction

Fetal structural abnormalities occur in approximately 3 % of pregnancies and are typically detected through ultrasonography, the most commonly used imaging method. Genetic analysis identifies genetic abnormalities in about 1 in 150 live births, 25 % of all miscarriages, and 50–60 % of stillbirths and first-trimester abortus [1]. In particular, the frequency of aneuploidy correlates with maternal age. For a period of time, standard karyotyping was considered the gold standard for detecting large structural anomalies (5–10 mB) such as polyploidy and aneuploidy [2]. However, standard karyotyping is insufficient for detecting copy number variants (CNVs), which are submicroscopic deletions and duplications involving small portions of chromosomes. This limitation has led to the increased popularity of CMA analysis in recent years [3]. It allows for the detection of CNVs as small as 50–100 kb and is widely used for conditions such as multiple congenital anomalies, developmental disorders, and autism spectrum disorders [4].

Chromosomal microarray analysis (CMA) utilizes next-generation sequencing (NGS) technology and is cost-effective. In fetuses with structural abnormalities detected by ultrasound, CMA provides an additional 6 % diagnostic yield compared to standard karyotyping [5]. However, it is limited in detecting inversions and translocations. With the advancement of next-generation sequencing (NGS), whole-exome sequencing (WES) has become valuable for diagnosing various diseases, particularly neurodevelopmental disorders, both during the prenatal and postnatal periods [6]. Its application in the postnatal period is less comprehensive compared to imaging modalities used in the prenatal period but is ideal for targeted examinations based on postnatal findings. Whole-exome sequencing (WES) utilizes a ‘larger sample size,’ which offers additional insights for classifying subgroups and may help identify pathogenic variants [7]. Targeted molecular testing can be conducted based on specific findings from imaging modalities such as USG or MRI. If targeted molecular testing yields normal results, there may still be non-genetic developmental birth defects or genetic disorders that are not detectable by conventional karyotyping and CMA.

In this study, we analyzed the results of conventional karyotype analysis, CMA, and WES applied to pregnant women, as well as their correlation with ultrasound findings.

## Materials and methods

This retrospective study was conducted at Eskisehir City Hospital, Turkey between 2020 and 2023. The study was approved by our Institutional Review Board. Fetal USGs were conducted on pregnant females referred to our clinic with suspected fetal anomalies and those with anomalies detected by targeted ultrasound. The ultrasounds were performed by a single perinatologist using a Voluson E8 (General Electric) device. Each anomaly detected by ultrasound was documented individually.

After diagnosis, all parents were given comprehensive counseling. Karyotyping and CMA analysis were presented to all fetuses with amniocentesis, CVS and cordocentesis. When no abnormalities were detected by karyotyping, CMA and WES was offered as an option. Demographic data and ultrasound findings of pregnant females with both normal and abnormal WES results were compared. Pregnant females for whom demographic data and ultrasound findings were unavailable were excluded from the study.

For CMA, the GRCh37 reference genome was examined using the Affymetrix Cytoscan Optima Suite method at 315k resolution. Duplications larger than 200 kb and deletions larger than 400 kb were deemed significant for prenatal diagnosis, according to ACMG guidelines. Smaller deletions and duplications were considered significant if accompanied by clinical findings. For whole-exome sequencing (WES) analysis, the DNBSEQ-G400 (MGI Tech Co., Ltd.) and the Twist Human Core Exome Kit (Twist Bioscience, San Francisco, CA, USA) were used.

SPSS 25.0 (IBM Corporation, Armonk, NY, USA) software was used for variable analysis. The data’s conformity to a normal distribution was assessed using the Shapiro–Wilk test, and the homogeneity of variance was evaluated with the Levene test. For comparing two groups based on quantitative variables, parametric tests were conducted using the Independent Samples *t*-Test with bootstrap results. Nonparametric comparisons were made using the Mann-Whitney *U* test with Monte Carlo simulation results. Fisher Exact, Fisher–Freeman–Halton and Linear-by-Linear Association tests were performed with the Monte Carlo Simulation results for the comparison of categorical variables. The Mc-Nemar test (Monte Carlo) was used to check the consistency between WES and Microarray values. Quantitative variables were expressed as mean (standard deviation) and median (minimum/maximum), while categorical variables were expressed as n (%). Variables were analyzed at a 95 % confidence level and a p-value of less than 0.05 was considered significant.

## Results

Twenty-six pregnant women with WES results meeting the inclusion criteria were divided into two groups: normal and abnormal. There was no statistically significant difference between the CMA results, invasive diagnostic indications, parity, gestational age, or risk levels in the screening tests of the 14 pregnant women with normal WES results compared to the 12 with abnormal WES results (see Table 1). Invasive diagnostic tests included amniocentesis and chorionic villus sampling (CVS) in 12 cases (46.2 %), and cordocentesis in two cases (7.7 %). The grouping of WES and microarray results into normal and abnormal karyotype is shown in Table 2.

**Table 1:** Distribution of descriptive characteristics of patients.

Variables 1	n (%)	Variables 2	n (%)
WES (positive)	12 (46.2)	WES (normal), microarray (abnormal)	8 (30.8)
Hyperechogenic bowel	6 (23.1)	Short rib	1 (3.8)
Clenched hand	4 (15.4)	Ambiguous genitalia	1 (3.8)
Forearm dysplasia	4 (15.4)	Aberrant right subclavian artery	1 (3.8)
Nuchal fold increase	3 (11.5)	Ductus venosus agenesis	1 (3.8)
Micromelia	3 (11.5)	Single artery single vein	1 (3.8)
Thoracic hypoplasia	2 (7.7)	Partial corpus callosum agenesis	1 (3.8)
Posterior urethral valve	2 (7.7)	Ascites	1 (3.8)
Intracardiac hyperechogenic focus	2 (7.7)	Encephalocele	1 (3.8)
Muscular VSD	2 (7.7)	Holoprosencephaly	1 (3.8)
Ventriculomegaly	2 (7.7)	Polycystic kidneys	1 (3.8)
CPC	1 (3.8)	Persistent right umbilical vein	1 (3.8)
Early-onset IUGR	1 (3.8)	Renal agenesis	1 (3.8)
CSP width	1 (3.8)	Lissencephaly	1 (3.8)
Pericardial effusion	1 (3.8)	Pyelectasis	1 (3.8)
Absence of gallbladder	1 (3.8)	Microcephaly	1 (3.8)
Invasive test indication (0/1/2)	1 (3.8)	18 (69.2)	7 (26.9)

	**Mean (SD.)**	**Median (min/max)**	

Gestation week, weeks	22.19 (4.41)	22 (12/32)	
Risk in screening test (×10^3^)	34.27 (98.12)	2.14 (1/476.19)	
Number of USG findings, n	1.92 (1.41)	2 (0/5)	
Parity, n	1 (1.26)	1 (0/5)	

SD, standard deviation; min, minimum; max, maximum.

**Table 2:** Analysis of patients according to WES and microarray results.

	WES (normal)	WES (positive)	p-Value
	(n: 14)	(n: 12)	
	n (%)	n (%)	
Microarray			0.344^a^
Normal	11 (78.6)	7 (58.3)	
Abnormal	3 (21.4)	5 (41.7)	
Process			0.061^d^
1	9 (64.3)	3 (25.0)	
2	0 (0.0)	2 (16.7)	
3	5 (35.7)	7 (58.3)	
Invasive test indication			0.826^d^
0	0 (0.0)	1 (8.3)	
1	10 (71.4)	8 (66.7)	
2	4 (28.6)	3 (25.0)	

	**Mean (SD)**	**Mean (SD)**	

Gestation week	22.29 (3.29)	22.08 (22.08)	0.090^c^

	**Median (min/max)**	**Median (min/max)**	

Parity	1 (0/5)	1 (0/4)	0.841^b^
Risk in screening test (×10^3^)	1.67 (1/178.57)	3.31 (1/476.19)	0.286^b^

^a^McNemar Test (Monte Carlo). ^b^Mann–Whitney *U* Test (Monte Carlo). ^c^Independent Samples *t*-Test (Bootstrap). ^d^Fisher–Freeman–Halton Test (Monte Carlo). min, minimum; max, maximum; SD, standard deviation.

After categorizing the WES results into normal and abnormal groups, the number of ultrasound findings for each group of pregnant females is shown in Table 3. The number of USG findings was higher in pregnant females with normal WES results. However, when comparing the USG findings within each group, no statistically significant differences were observed.

**Table 3:** Analysis of USG findings according to WES results.

	**WES (normal)**	**WES (positive)**	**p-Value**
	**(N: 14)**	**(N: 12)**	
	**Median (min/max)**	**Median (min/max)**	

**Number of USG findings**	**2 (1/5)**	**1 (0/3)**	**0.012** ^a^
	**n (%)**	**n (%)**	

Number of USG findings			0.010^c^
0	0 (0.0)	3 (25.0)	
1	4 (28.6)	5 (41.7)	
2	4 (28.6)	3 (25.0)	
3	2 (14.3)	1 (8.3)	
4	2 (14.3)	0 (0.0)	
5	2 (14.3)	0 (0.0)	
Hyperechogenic bowel	5 (35.7)	1 (8.3)	0.170^b^
Clenched hand	4 (28.6)	0 (0)	0.100^b^
Forearm dysplasia	4 (28.6)	0 (0)	0.100^b^
Microcephalia	0 (0)	1 (8.3)	–
CPC	0 (0)	1 (8.3)	–
Nuchal fold increase	3 (21.4)	0 (0)	–
Pyelectasis	0 (0)	1 (8.3)	–
Ventriculomegaly	1 (7.1)	1 (8.3)	–
Early-onset IUGR	1 (7.1)	0 (0)	–
Intracardiac hyperechogenic focus	2 (14.3)	0 (0)	–
Muscular VSD	1 (7.1)	1 (8.3)	–
CSP width	1 (7.1)	0 (0)	–
Posterior urethral valve	2 (14.3)	0 (0)	–
Pericardial effusion	1 (7.1)	0 (0)	–
Absence of gallbladder	1 (7.1)	0 (0)	–
Thoracic hypoplasia	1 (7.1)	1 (8.3)	–
Short rib	1 (7.1)	0 (0)	–
Micromelia	1 (7.1)	2 (16.7)	–
Ambiguous genitalia	1 (7.1)	0 (0)	–
Aberrant right subclavian artery	0 (0)	1 (8.3)	–
Ductus venosus agenesis	1 (7.1)	0 (0)	–
Single artery single vein	1 (7.1)	0 (0)	–
Partial corpus callosum agenesis	1 (7.1)	0 (0)	–
Ascites	1 (7.1)	0 (0)	–
Encephalocele	0 (0)	1 (8.3)	–
Holoprosencephaly	1 (7.1)	0 (0)	–
Polycystic kidneys	0 (0)	1 (8.3)	–
Persistent right umbilical vein	1 (7.1)	0 (0)	–
Renal agenesis	0 (0)	1 (8.3)	–
Lissencephaly	0 (0)	1 (8.3)	–

^a^Mann–Whitney *U* Test (Monte Carlo). ^b^Fisher–Freeman–Halton Test (Monte Carlo). ^c^Linear-by-Linear Association. min, minimum; max, maximum. WES, whole exome sequencing; CPC, choroid plexus cyst; IUGR, intrauterine growth restriction; VSD, ventrıculer septal defect; CSP, cavum septum pellucidum.

## Discussion

With the advancement of NGS technology, CMA and WES analyses are becoming increasingly common in clinical practice, particularly in prenatal and postnatal settings. In this study, we examined the correlation between the results of CMA and WES analyses and ultrasound findings in pregnant women, who had previously received a normal karyotype analysis.

With the introduction of CMA and WES technology, the detection of genetic anomalies has significantly increased in both prenatal and postnatal periods. A large cohort study of 610 fetuses conducted by Lord et al. found a genetic anomaly rate of 8.5 % [8]. Additionally, clinically significant variants were identified in 12.5 % of cases. The study found a correlation between genetic anomalies and abnormalities in the cardiac, multi-system, and skeletal systems detected by ultrasound. The authors concluded that incorporating WES with CMA analyses could enhance diagnostic capabilities and be beneficial for subsequent pregnancies. Although WGS is theoretically superior to WES, it is recommended to use CMA and WES together until WGS is more conclusively validated. In our study, CMA and WES were performed in cases where conventional karyotype analysis results were normal.

Indications for genetic examination are often based on USG findings [9]. Yates et al. conducted a WES analysis of 84 deceased fetuses [10]. Of these analyses, 20 % were positive, 45 % were classified as possible, 9 % identified only candidate gene variants, and 26 % were negative. The most frequently reported abnormalities detected by ultrasound were CNS anomalies, hydrops/edema, and cardiovascular abnormalities. A smaller study by Leung et al. included 33 fetal samples [11]. Multiple system anomalies were detected in approximately half of the fetuses. In fetuses with normal CMA results, 9.1 % had findings detected by WES, and 18.2 % had variants of unknown significance (VUS). The study by Zhou et al. evaluated the results of WES, WGS, and CMA in 110 pregnant women [12]. WES analysis was performed on 102 cases that were reported as negative in CMA examination. Genetic anomalies were detected in 13 cases (12.7 %) during this analysis. All genetic anomalies found in 22 cases (19.8 %) with CMA+WES results were also detected by WGS. Additionally, the analysis provided diagnostic opportunities for intrauterine infections and balanced translocation cases. In our study, the most frequently observed ultrasound findings in the WES-positive group were, in order, hyperechoic bowel, clenched hand, and forearm dysplasia. However, no cases of intrauterine infection were observed. A result that contradicts the literature is the finding that WES-negative pregnancies had more USG findings compared to WES-positive pregnancies. We attribute this discrepancy to the small number of cases in our study.

Quick results are crucial for genetic analysis in cases where termination may be necessary. As the fetus grows during the process, it is important to act swiftly in the event of a possible termination. For instance, FISH analysis is quite useful in this context. The study by Zhou et al. indicated that WGS has a superior turnaround time (18 ± 6 days) compared to that for CMA+WES (31 ± 8 days). Therefore, WGS is considered a potential alternative to CMA+WES. In our study, the turnaround time was not calculated, whereas Drury et al. conducted WES analysis on 24 pregnancies. This study identified genetic anomalies in five cases (21 %). These cases included Milroy disease, hypophosphatasia, achondrogenesis type 2, Freeman-Sheldon syndrome, and Baraitser–Winter syndrome.

In classifying anomalies detected during pregnancy monitoring, it is crucial to distinguish between single vs. multiple system anomalies and to identify which system the anomaly affects. Among organ systems, the cardiac system is associated with the highest increased risk of anomalies. Additionally, the presence of multiple system anomalies further increases the risk. Fu et al. studied approximately 4,000 pregnant females in their research [13]. This study found that genetic anomalies were detected in 18.2 % of cases. Pathogenic CNVs were identified in 8.2 % of pregnant women with normal karyotype results from conventional analysis. WES examination was performed on 196 fetuses with normal CMA results, revealing an abnormal phenotype in 24 % of cases. Anomalies were detected in 22.3 % of pregnancies with single malformations and in 30.8 % of cases with multiple malformations.

In the review prepared by Mellis et al., 66 studies evaluating a total of 4,350 pregnant females were examined [14]. WES analysis provided an additional diagnosis in 31 % of cases where structural anomalies were reported as having a normal karyotype by CMA analysis. Among the structural anomalies, skeletal system anomalies were found in 53 % of cases, neuromuscular anomalies in 37 %, and multi-system anomalies in 29 %. Increased NT and isolated gastrointestinal anomalies (2 %) had the least diagnostic value. Reilly et al. evaluated 21 studies involving approximately 2,000 cases of congenital heart disease (CHD). Unlike other studies, they compared all types of CHDs, isolated CHDs, and extra-cardiac anomalies associated with CHD. The frequencies of these anomalies were found to be 17.4 , 9.3, and 35.9 %, respectively. In our study, WES-positive patients had two intracardiac hyperechogenic foci, muscular VSD, ventriculomegaly, and one pericardial effusion. In WES-negative cases, we identified one aberrant right subclavian artery (ARSA) and ductus venosus agenesis.

More studies with large sample sizes, randomized controlled design, and multicenter collaboration are needed to evaluate the most appropriate method for patients with various degrees of pregnancy. We also lack long-term follow-up. And we are in continuous follow-up.

## Conclusions

We believe that combining CMA and WES analysis is a necessary and valuable method for pregnant women with normal karyotype results from conventional karyotype analysis. Our ultrasound results are not consistent with the current literature and need to be reassessed with a larger patient cohort to align with existing findings.
